# Effect of Mining Activities in Biotic Communities of Villa de la Paz, San Luis Potosi, Mexico

**DOI:** 10.1155/2014/165046

**Published:** 2014-01-30

**Authors:** Guillermo Espinosa-Reyes, Donaji J. González-Mille, César A. Ilizaliturri-Hernández, Jesús Mejía-Saavedra, V. Gabriela Cilia-López, Rogelio Costilla-Salazar, Fernando Díaz-Barriga

**Affiliations:** ^1^CIACYT-Facultad de Medicina, Universidad Autónoma de San Luis Potosí, Avenida Sierra Leona No. 550, Lomas 2da Sección, 78210 México, SLP, Mexico; ^2^Life Sciences Division, Universidad de Guanajuato, Campus Irapuato-Salamanca, Carretera Salamanca-Valle de Santiago Km. 3.5 + 1.8, Palo Blanco, 36885 Salamanca, GTO, Mexico

## Abstract

Mining is one of the most important industrial activities worldwide. During its different stages numerous impacts are generated to the environment. The activities in the region have generated a great amount of mining residues, which have caused severe pollution and health effects in both human population and biotic components. The aim of this paper was to assess the impact of mining activities on biotic communities within the district of Villa de la Paz. The results showed that the concentrations of As and Pb in soil were higher than the national regulations for urban or agricultural areas. The bioavailability of these metals was certified by the presence of them in the roots of species of plants and in kidneys and livers of wild rodents. In regard to the community analysis, the sites that were located close to the mining district of Villa de la Paz registered a lower biological diversity, in both plants and wild rodents, aside from showing a change in the species composition of plant communities. The results of this study are evidence of the impact of mining on biotic communities, and the need to take into account the wildlife in the assessment of contaminated sites.

## 1. Introduction

Mining is one of the most important industrial activities worldwide. It is estimated that there are at least 10,000 mining industries and more than 20,000 mining sites, mineral processing plants, and smelting [1]. Nevertheless, it is considered to be a productive activity with a high environmental impact because in its different stages (exploration, extraction, and processing) it generates numerous adverse effects, as well as a great amount of residues, which could cause water, soil, and sediment pollution [2, 3]. The generation of residues (tailing dams, deposits, and slag) is one of the most notorious environmental impacts in the mining activity, for they are considered to be the source of heavy metals such as cadmium (Cd), chromium (Cr), copper (Cu), lead (Pb), zinc (Zn), and metalloids such as arsenic (As) [4]. This pollution source represents in a major or minor way a risk, for both human population (health) and for biota (ecological) living in the study site [5, 6].

Mining brings as a consequence diverse types of impact that could affect the composition and structure of the biotic communities (richness, abundance, diversity, dominance, trophic relationships, etc.); among the most evident impacts we can find (1) elimination of vegetation, which alters the availability of food and shelter for wild animals and (2) the toxic effects in the health of organisms derived from the presence of heavy metals [7–10].

In Mexico around 5,036,836,611.54 tons per year of metals such as silver (Ag), gold (Au), Cu, Cd, lead, and Zn are extracted, which represents 1.3% of the gross domestic product, showing that mining is an important economic activity in our country [11].

The mining district of Santa María de la Paz is located between the municipalities of Villa de la Paz and Matehuala in the state of San Luis Potosí. A skarn deposit of Pb-Zn-Ag (Cu-Au) (metamorphic rocks made of silicates of calcium (Ca), iron (Fe), and magnesium (Mg) derived from a protolith limestone and dolomite in which great quantities of aluminum (Al), Fe, and Mg have been introduced) is found in this district.

The main activity in Villa de la Paz is the extraction of fluorite, Zn, Ag, Cu, Pb, bentonite, Au, clay, silica, limestone, and salt. For the past few years 2007–2012 246,665.36 ton of Au, Ag, Cd, Cu, Pb, Zn has been extracted [11]. This activity has been taking place for the past 200 years, and it has generated a great amount of waste (tailings and deposits), which lies in open air and is exposed to environmental weathering; it represents a risk of environmental pollution.

Several studies have been done in that mining region to determine whether risk to human health and diverse biological components exists. Among the studies that have been done, there are those regarding environmental characterization [12–17]; in some of these, it has been proven that risk for the health of human populations in the area due to the exposure to heavy metals and As exists [18–24]. On the other hand studies regarding exposure and effects on diverse species of flora and fauna have also been done [25–27].

Heavy metal accumulation in plants has multiple direct and indirect effects on plant growth and alters many physiological functions by forming complexes with O, N, and S ligands. They interfere with membrane functioning, water relations, protein metabolism, and seed germination [28]. Impediment of proper absorption and essential element transport in plants, metabolic alterations, decrease and inhibition of adequate growth, reproductive alterations, wilting, chlorosis, dehydration, mortality, and photosynthesis inhibition are among the main reported effects on plants due to heavy metal exposure [29–31].

Regarding the effects of mining activities on plant communities, the presence of different concentration levels of metals such as Cd, Cu, Se, and Zn in soil and water has been reported and is known to reduce the diversity and abundance in plant communities [32, 33].

The study of diversity and abundance among species has been commonly used as an indicator of biotic integrity in different types of ecosystems [34, 35]. Some studies have used the diversity and structure of communities (plants and/or animals) to demonstrate the effect of exposure to a certain pollutant such as pest-control substances, metals, herbicides, chemical wastes, residual water discharge, to name a few [32, 36–41].

The study done on wild rodents and small mammals in polluted sites has been focused on evaluating the bioaccumulation and sublethal effects (DNA damage, oxidative stress, etc.) [25, 42–50]. These kinds of projects are important but are generally not very ecologically relevant. In what concerns studies in superior levels of biological organization in Rusia Kataev et al. [51] registered that the population densities of rodents that are found close to where the mining activity takes place are lower in comparison to the ones found farther away.

The objective of this study was to evaluate the impact of the mining activity on biotic communities in the mining district of Villa de la Paz. In order to do this the determination of the heavy metals and As was done in soil and roots of plants as well as the estimation of parameters in the communities of plants and rodents.

## 2. Materials and Methods 

### 2.1. Study Site

The mining district of Santa Maria de la Paz is located on 23°41′ longitude N and 100°38′ latitude W. Within this district we can find Villa de la Paz, which has been exploited for over 200 years. The predominant weather in the region is mild semidry with rains in the summer [52]. The average annual temperature is between 12° and 18°C; the total annual precipitation varies from 400 to 600 mm [53]. Haplic and calcic xerosols are the dominant soils, which are the typical types of soil in semiarid regions [53]. The main types of vegetation are the xerophytic scrubland and the gypsophyllous grassland [54]. The main economic activities in the region are mining, agricultural production, livestock production, and tourism [53, 55].

### 2.2. Selection of Sampling Sites

**Figure 1:**
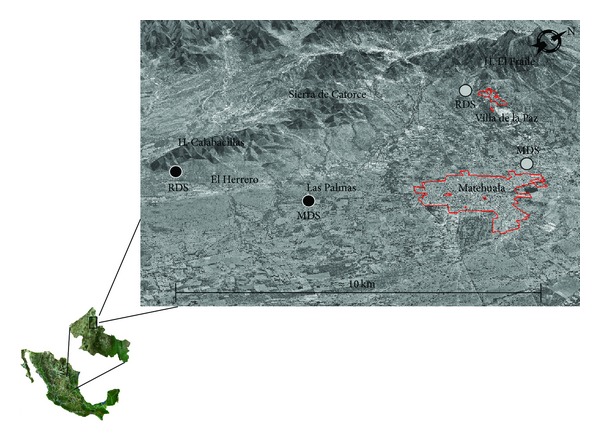
Location of the sampling sites in the mining region of Villa de la Paz, San Luis Potosi, Mexico. Contaminated sites (gray circles); reference sites (black circles). RDS: rosettophyllous desert scrub; MDS: microphyllous desert scrub.

The main types of dominant vegetation selected within the area of study were the microphyllous desert scrub (MDS) and the rosettophyllous desert scrub (RDS); a sampling site was chosen within in each of these, considering the areas with the highest arsenic and metal concentrations [15, 25]. Aside of this, two sites within a reference zone were selected (with similar exposure, relief, lithology, altitude, and condition) to make a comparison with the polluted zone. This zone is located, approximately 10 km south of the impacted zone (Figure 1).

Plant, root, and soil samplings took place in each of the selected sites during the months of May and June of 2004.

### 2.3. Environmental Monitoring

The quantification of As, Cu, Pb, and Zn was done in plant species, in order to measure the concentrations to which they were exposed. Fifty samples from the superficial soil were obtained (0 to 10 cm of depth) in each of the sampling sites, to obtain a total of 60 soil samples. The samples were collected using the same transects that were used for the plant sampling (see under), five points were established every 5 m within the sampling areas, starting from the origin of the line (0 m) and up to 20 m [56]. In each of the points, 10 cm^3^ blocks of soil were extracted using a shovel and were put in polyethylene bags, so they could be transported and analyzed elsewhere.

### 2.4. Monitoring of Plant Species

The plant sampling was done through the point quadrats method [57] and three transects were traced perpendicularly to the slope in each of the sites (in a southeast-northeast direction, with an average length of 20 m) in order to obtain the frequency, the basal area, the density, and the value of importance of the plant species [58, 59].

In order to verify if absorption was happening in the roots, the quantification of As, Cu, Pb, and Zn had to be done. For this process, individuals were selected from the four dominant species of plants of each type of vegetation present in both the contaminated and the reference site. The selected species in the RDS were *Jatropha dioica *(Jadi),* Karwinskia mollis*  (Kamo),* Agave lechuguilla *(Agle), and *Dyssodia acerosa *(Dyac). The ones on the MDS were *J. dioica *(Jadi),* Larrea tridentata *(Latr),* Parthenium incanum *(Pain), and* Zinnia acerosa *(Ziac). Five individuals of each of these species were collected, except for *D. acerosa*, for which 10 individuals had to be taken so a composite sample could be done, because its roots are very small. All the collected plants were fully extracted, the aerial part (foliage) was eliminated, and the roots were collected in polyethylene bags for easier transportation for analysis.

### 2.5. Rodent Monitoring

Rodents were captured alive using Sherman traps, only in the MDS. The capture was done two nights in a row, with two repetitions per plot of land, in six different outings, which totaled 960 night-traps. All six outings for the capture and recapture of rodents were done during the period between September 2005 and May 2007. During the period of study the existing assumptions established by Seber [60] where complied with for the study of open populations, as well as getting to know the biology of all the species of rodents within the area of study [59]. A mixture of oats and vanilla was used as bait. The species of the captured rodents were determined as well as different morphometric parameters such as total length, of the tail, the body, the back legs, and the ear. Their sex was recorded and all individuals captured were marked [61, 62]. For the collection of plants and the capture of rodents we have the scientific collector permit FAUT-262 issued by Secretaría de Medio Ambiente y Recursos Naturales (SEMARNAT).

### 2.6. Parameter Estimates at a Community Level

The Shannon-Wiener index (*H*′) was used to determine the diversity of both plant and rodent species present on the sites: *H*′ = −∑_*i*=1_^*s*^(*Ni*/*N*)ln⁡⁡(*Ni*/*N*) [58, 63].

As for plant communities, all evaluated attributes were determined according to the vegetative stratus and for the total vegetation.

### 2.7. Analysis of As and Metals in Soil and Roots

The soil samples of roots and soil were put through a process of acid digestion for the quantification of As, Cu, Pb, and Zn. The digestion of the soil was done in a microwave oven (CEM MDS-2000) using nitric acid (HNO_3_), and for the roots the digestion had to be done using a heating plate with HNO_3_ and perchloric acid (HClO_4_).

The quantification of heavy metals was done using spectrophotometry of atomic absorption (EAA). Standard Reference Materials (SRM) which were certified by the National Institute for Standards and Tests of the United States of America (NIST) were analyzed, as a strategy for quality control. The employed SRM were the SRM 2710 for trace analysis in soils and SRM 1547 for plants. The percentage of recovery for both ranged from 80 to 104%.

### 2.8. Statistical Analysis

A Chi Square test, using a contingency chart of 2 × 2, had to be done in order to evaluate the differences in the composition of flora on the sites [64]. A two-sample *t*-test was used in order to compare the diversity (Shannon-Wiener index) [65, 66]. For the comparison of metal concentrations, the Kruskal-Wallis test in soil and the Mann-Whitney *U* test in plants were used. The level of statistical significance was considered at *P* < 0.05 for all tests. Statistical analysis was performed with STATISTICA (version 12.0, StatSoft Inc.). Diversity-abundance models were done in order to extract patterns of relative species abundances without reducing information and to make a comparison between reference and contaminated sites [66].

## 3. Results and Discussion 

### 3.1. Metals and As in Soil

In Table 1 the concentrations of the quantified metals in the superficial soil samples classified according to the type of vegetation are shown. For both types of vegetation, a statistically significant difference was found (*P* < 0.05) between the concentrations of the contaminated and reference site. In the case of RDS, As turned out to be five times higher, Cu 14 times, Pb 12 times, and Zn three times more than in the reference site. The decreasing order of concentration found in the contaminated site was Cu > As > Pb > Zn compared to the reference site which was Zn > As > Pb > Cu. For the MDS it was found that As was 344 times higher, Cu 27 times, Pb 52 times, and Zn 40 times more than the value from the reference site. The order of concentration on the contaminated site was As > Zn > Pb > Cu and for the reference site it was Zn > Cu > Pb > As.

In Table 1 the concentrations of As and Pb which surpass the maximum limits allowed according to the NOM-147-SEMARNAT-2004 are shown. Thresholds for Cu and Zn do not exist in Mexican regulations, which is the easy why the parameter established by the Canadian guides for environmental protection and human health had to be used as comparison; it was found that Cu exceeds the protection limits in both types of vegetation, while Zn was found to have exceeded the protection limits only in the MDS contaminated site [67]. This information concurs with reported results from other authors.

The registered differences in the concentrations of metals in soil among the contaminated and the reference sites confirm the impact of mining activity reported by Razo et al. [15]. In the contaminated sites, higher concentrations were found in the MDS than in the RDS; these differences can be attributed to the topographic location of the sites. The RDS site is found on the slope of the hill “El Fraile” which favors the movement of metals and As to lower areas due to run off. The MDS site is found in the valley, which probably explains why a higher concentration was reported. These results coincide with what was registered by Razo et al. [15], who reported lower concentrations in the hillside areas (As 731 mg/kg; Cu 206 mg/kg; Pb 433 mg/kg; and Zn 2351 mg/kg), compared to the ones in the valley (As 10459 mg/kg; Cu 1023 mg/kg; Pb 1560 mg/kg; and Zn 5205 mg/kg).

Regarding the concentrations that were registered in the reference area, these were found to be within the range of what some authors registered as being basal (As 1–40 mg/kg; Cu 1–140 mg/kg; Pb 10–70 mg/kg; and Zn 5–125 mg/kg) [32, 68, 69].

### 3.2. Metals and As in Roots

The quantification of the content of metals in the roots of plants was done for the purpose of determining if they could be absorbed by plants. The concentrations of As, Cu, Pb, and Zn, found in the roots of the selected species according to their vegetation type, are shown in Table 2. In the RDS, no differences were found between the contaminated and the reference site for any metal in the *K. mollis *specie (*P* > 0.05); however for the *A. lechuguilla* and *J. dioica *species differences were found (*P* < 0.05) (with the exception of Zn in* A. lechuguilla* and Pb in *J. dioica*). In the MDS a statistically significant difference was found (*P* < 0.05) between the concentrations of the four species from the contaminated and reference sites (with the exception of Zn en *J. dioica*). Zn was the element that was absorbed in the highest amounts (with the exception of *J. dioica* where it was Pb).

According to these results, it can be stated that metals and As present in the area are available for the plant species; however, not all plants have the same method for absorption and/or translocation. It has been observed that plants develop different resistance mechanisms when exposed to high metal concentrations, which makes them absorb metals in a differential way [70–72]. In this regard Machado-Estrada et al. [27] did the evaluation of the translocation of metals and As in plant species (*A. lechuguilla, P. incanum,* and *Z. acerosa*) of the same sites that we evaluated in Villa de la Paz and the reference area. They state that there is more translocation towards stems and leafs in recollected plants in the polluted sites, which shows the bioavailability of the metals in these species of plants.

Added to this, the absorption of metals in plants depends mainly on bioavailability of metals and on the intrinsic characteristics of each species. Several authors have reported that bioavailability of metals depends on factors such as condition of soil, pH, and the chemical form [73–75]. According to them, the differences in the absorption by the plants of the region can be attributed to the possible development of resistance mechanisms towards metals (e.g., evasion) and to the presence of the different chemical forms of metals generated in the mining processes that have been reported in the area by Razo et al. [15].

### 3.3. Metals and As in Rodents

Jasso-Pineda et al. [25] evaluated the concentrations of As, Cd, and Pb in livers and kidneys of the rodents that were captured in the mining district of Villa de la Paz and in a reference site. Our study was done jointly (sampling sites and seasons) with the previously mentioned study. On that basis the concentrations of metals in the tissues of rodents registered in the article by Jasso-Pineda et al. [25] can be directly compared to the concentrations of metals in soil and the roots of plants. The levels of registered metals, in all cases, were significantly higher in the contaminated site compared to the reference site.

### 3.4. Plant Communities

As it is shown in Table 3, the richness of the species was similar between the contaminated and reference sites of the RDS (20 and 19 species, resp.) (*P* > 0.05); unlike the MDS in which the reference site showed a major richness (32 species) than the contaminated site (13 species) (*P* < 0.05).

Regarding the total diversity of the RDS (Table 3), a statistically significant difference was registered (*P* < 0.05), between the contaminated and the reference site. There was also a significant difference for the bushy stratus (*P* < 0.05), but not for the herbaceous one (*P* > 0.05).

Aside from this, it was determined that the contaminated site has a higher diversity than the reference site (Table 3), which is given by the highest abundance of individuals in the bushy stratus. The total diversity of the MDS, as well as the stratus (Table 3) of the contaminated site, was higher (*P* < 0.05) than the ones from the reference site.

**Figure 2:**
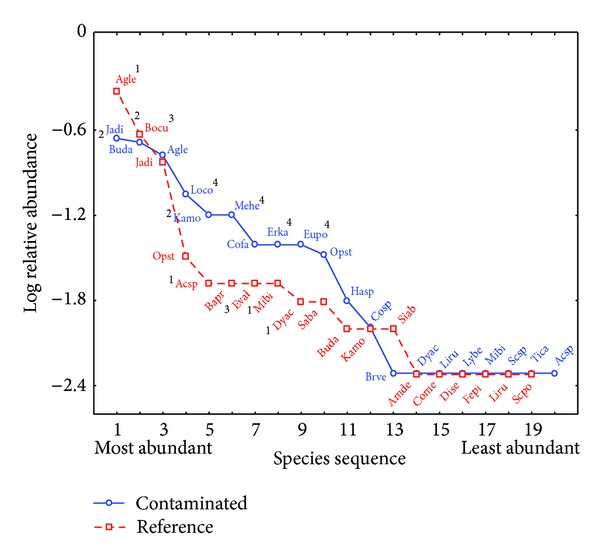
Abundance-diversity model from the rosettophyllous desert scrub (RDS). 1: species with the greatest abundance in the reference site, 2: species with a greater abundance in the impacted site, 3: species only present in the reference site, and 4: species only present in the impacted site (see Table 4 for the acronyms).

The elements of diversity are richness and equity of species. In the case of RDS in both sites the richness was the same (Table 3); likewise the relative abundance (number of individuals per species) was similar in both communities, just not with the same species (Figure 2), which explains why communities do not show differences in the value of the diversity index. In the contaminated site, a larger diversity of species was found in the bushy stratus (Table 3), because the species present in this site had a greater abundance of individuals (Figure 2). This difference among the diversity of individuals could be attributed to the change in species, because this new species could have lower environmental requirements and/or less competition in comparison with the ones that were not registered, which allows them to have a greater number of individuals [66].

**Figure 3:**
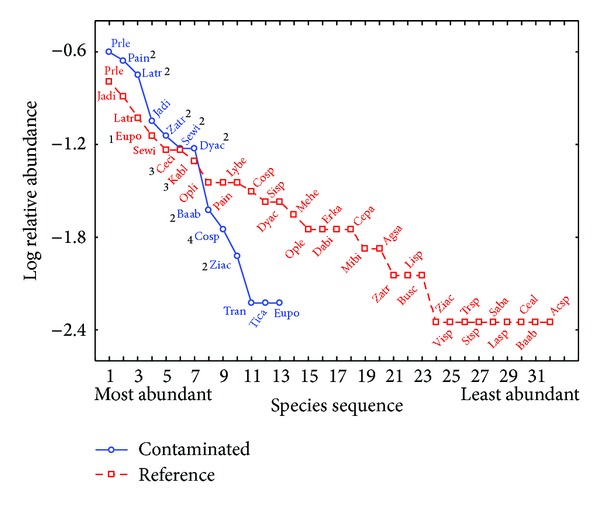
Abundance-diversity model from the microphyllous desert scrub (MDS). 1: species with the greatest abundance in the reference site, 2: species with a greater abundance in the impacted site, 3: species only present in the reference site, and 4: species only present in the impacted site (see Table 4 for the acronyms).

In the case of MDS the way communities responded to metals was by reducing the diversity of species (Table 3), which could be caused by a difference in sensibility towards pollutants among the plants species that are present; this way more sensitive species are replaced with tolerant species or are eliminated completely (Figure 3) [66, 76].

In the RDS regarding the composition of species in the sites, it was found that the contaminated and the reference sites share 9 species; 11 species are only present in the contaminated site and 10 only registered in the reference site. A statistically significant difference was found (*P* < 0.05) among the sites (Table 4).

In the case of MDS 11 species were registered and they were present in both sites, 3 species were registered only on the contaminated site and 21 of them only on the reference site. The difference regarding composition in both sites turned out to be statistically significant (*P* < 0.05) (Table 4).

The diversity-abundance models of the RDS are shown in Figure 2, where similarities in the behavior between the contaminated and the reference site can be seen, meaning that both sites had the same amount of both abundant species and not very abundant ones. In the case of MDS (Figure 3) it can be observed that the contaminated site behaves completely different compared to the reference site, because there were mostly abundant species. Abundant and not very abundant species were registered in the reference site.

In the RDS, a change between the floral composition of the communities in the contaminated and the reference zone was found (Table 4), although it kept the same richness (Table 3) and a relationship is still being maintained regarding the proportion among the abundance of species (Figure 2), which was not the case for the MDS where aside from the change in species (Table 4) the richness showed a contrast between both sites (Table 3). In the contaminated site a higher number of dominant species were present, and there was an absence of species with low abundance (Figure 3). With the results it became evident that the change in floral composition was one of the effects of metals in the communities of the area, which is similar to what has been reported in other environments [7–9].

Luoma [77] arguments that the presence of species tolerant to pollution in a community is a strong evidence that a pollutant is causing adverse effects over it. Some of the species that were only found in the contaminated zone of the RDS (*B. veronicaefolia, E. karvinskianus, L. tridentata,* and *P. laevigata*) have previously been registered as being accumulative and tolerant to metals [78–81].

The presence of tolerant species and the absence of sensitive ones, the increase or decrease in the abundance of species, the change in their composition are the main effects that were found in plant communities within the area of Villa de la Paz; these effects are causing a change in the structure of their communities, which could lead to an imbalanced ecosystem, and it will be reflected in the alterations of its properties such as productivity and the biogeochemical cycles. The plant communities in the MDS are at higher risk than the communities in the RDS, because of the changes on their structure and the loss of diversity, which can lead to an imbalance of their ecosystem properties. Aside of this, if the impacts towards the environment continue, the communities from the RDS could present this same risk.

One of the first visible symptoms of the environmental stress due to contamination is the changes in vegetation, the latter being a component of an ecosystem which fully reflects the features of its site. The changes in the plant communities also represent a direct risk for the animal communities that depend on them. As a result, plant communities are a good indicator of the changes in the site. Hence, research on the changes in vegetation, as well as changes in the structure of plant communities and their dynamics, provides the necessary information not only for assessing the degree of contamination and degradation of the environment, but also for the measurement of the decrease in its production potential [82].

### 3.5. Wild Rodent Communities

During the six outings for the systemic capturing-recapturing of rodents, a total of 77 rodents were captured (43 from the reference site and 34 from the polluted site).

The sampling period took place from September 2005 to May 2007. In the reference site, six species belonging to two different families were captured, Heteromyidae and Muridae, and in the contaminated site two species belonging to the Heteromyidae family were found (Table 5).

**Figure 4:**
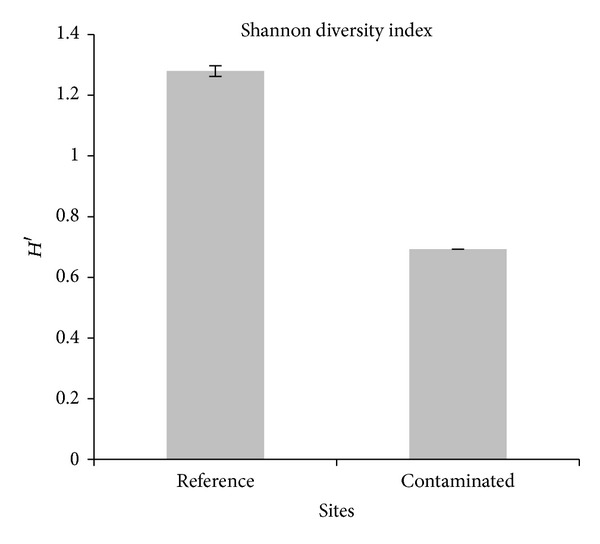
Diversity of rodents in the mining site of Villa de la Paz and the reference zone.

Just like in the plant communities, diversity is made up of the number of species (richness) and the number of individuals of each species (equity). In Figure 4 how the rodent community diversity in the reference site (*H*′ = 1.28) is greater than that in the contaminated site (*H*′ = 0.69) is shown; it is likely that this decrease in the diversity is actually a response from communities towards metals, which could be attributed to a difference in sensitivity towards contaminants among species. This way, sensitive species are replaced for tolerant species or they are definitely eliminated [66, 74].

One alternative is that the concentrations of metals can first of all affect the species of plants that are used as source of food for distinct species of rodents, and with the absence of species to feed upon, rodents will emigrate to other areas.

There are no similar studies in communities of rodents with which we can compare the obtained results on this research.

Species richness has been employed as indicator of ecological integrity (ecosystem distress syndrome); reduced species richness is one of the most consistent responses to assess effects of contaminants and also has high social relevance. Diversity indices provide important information about rarity and commonness of species in a community; they have been employed as integrative indicator. The ability to quantify diversity in this way is an important tool for biologists and ecotoxicologist trying to understand community structure and effects derivatives of anthropogenic activities in biomonitoring studies [66].

## 4. Conclusions

The concentrations of As and Pb exceed the maximum allowed limits established by the NOM-147-SEMARNAT-2004 and by the Canadian guides for environmental protection. Metals and As in the mining district of Villa de la Paz are bioavailable, because concentrations of them were found in roots and organs of wild rodents [25]. Regarding the community level analysis a change in the plant composition was registered, as well as a loss in diversity of the plant communities and rodents, high concentrations of metals present in plants, the tissues of rodents, and the roots of plant species, and the registered effects on a molecular level in rodents from the contaminated site [25], all strong evidence of the existent risk in the biotic communities of Villa de la Paz, San Luis Potosi, caused by the mining activities that take place there. However, there is also a possibility that this loss of diversity is due to the continuous fragmentation of the habitat and regional stressors (e.g., climatic change, desertification, and drought) in the municipalities of Villa de la Paz and Matehuala.

It is also important to mention that risk assessments are made in Mexico often neglects the assessment of biota (some components), which represents a severe problem because they are an essential part of the ecosystem and if the biotic components are affected at some time will be reflected in the health of the human population. In the northern part of Mexico and particularly in San Luis Potosi there are various mining areas (Villa de Ramos, Charcas, and Cedral), with similar characteristics as Villa de la Paz. The results of this study will allow the selection of sites and species in order to incorporate them in a medium and long range monitoring program on the effect of metals and As for the region.

## Figures and Tables

**Table 1:** Concentrations (mg/kg) of As, Cu, Pb, and Zn in soil by vegetation type.

Vegetation types	Site	As	Cu	Pb	Zn
RDS	Reference	46.8	16.3	16.8	57.7
		(12.0–23.0)	(10.0–20.0)	(23.8–97.6)	(45.0–125.0)
	Contaminated	222.1*	231.9*	204.3*	175.4*
		(125.8–329.3)	(155.0–382.5)	(117.9–487.1)	(125.0–275.0)
MDS	Reference	23	25.6	23.5	87.3
		(18.3–38.5)	(22.5–30.0)	(15.9–31.2)	(57.5–280.0)
	Contaminated	7902.6*	703.7*	1228.2*	3513.5*
		(502.0–17325.0)	(292.5–1080.0)	(428.1–2226.8)	(505.0–6325.0)
NOM-147-SEMARNAT/SSA1-2004 agricultural/residential land		22	—	400	—
CEQG agricultural land		12	63	70	200

The values represent the mean and range. RDS: rosettophyllous desert scrub; MDS: microphyllous desert scrub. *Statistically different concentrations (*P* < 0.05) in relation to the reference site; —: not mentioned.

**Table 2:** Concentrations (mg/kg) of As, Cu, Pb, and Zn, in roots, by species and vegetation type.

Vegetation types	Site	Specie	As	Cu	Pb	Zn
RDS	Reference	**Kamo**	1.6	2.0	0.3	9.3
			(1.4–1.9)	(1.5–2.8)	(0.2–0.4)	(5.0–14.1)
		**Agle**	0.5	2.8	0.5	8.9
			(0.1–1.0)	(2.3–3.5)	(0.4–0.6)	(5.8–13.0)
		**Jadi**	0.7	3.2	0.5	11.2
			(0.2–1.1)	(2.5–4.8)	(0.2–1.0)	(7.5–14.8)
		**Dyac**	0.7	10.0	22.3	1.0
	Contaminated	**Kamo**	1.8	1.8	0.4	5.6
			(0.7–2.3)	(0.2–2.8)	(0.3–0.5)	(4.5–7.0)
		**Agle**	1.1*	4.5*	1.7*	8.4
			(0.8–1.6)	(3.3–6.1)	(0.7–3.6)	(6.4–13.0)
		**Jadi**	2.6*	7.3*	0.9	37.8*
			(1.1–4.7)	(3.0–10.5)	(0.3–2.0)	(13.5–52.0)
		**Dyac**	1.1	11.3	16.0	2.1

MDS	Reference	**Latr**	0.3	6.0	0.3	12.5
			(0.1–0.7)	(3.9–7.4)	(0.2–0.5)	(8.9–20.5)
		**Pain**	0.3	3.3	0.6	14.0
			(0.2–0.5)	(2.5–3.6)	(0.4–0.7)	(10.8–17.5)
		**Jadi**	1.1	1.8	0.1	6.0
			(1.0–1.4)	(1.5–2.0)	(0.02–0.3)	(4.5–7.8)
		**Ziac**	1.6	2.8	4.5	6.7
			(0.8–2.3)	(1.5–7.5)	(2.8–5.6)	(5.3–8.5)
	Contaminated	**Latr**	4.3*	10.6*	1.3*	38.5*
			(1.4–14.1)	(8.0–18.1)	(0.4–4.1)	(12.8–129.3)
		**Pain**	5.1*	14.0*	2.2*	23.1*
			(3.2–6.2)	(9.5–19.8)	(1.4–2.5)	(17.8–30.0)
		**Jadi**	22.0*	13.4*	2.3*	5.4
			(1.9–43.9)	(8.5–20.5)	(1.4–3.2)	(5.0–6.3)
		**Ziac**	15.2*	12.0*	365.5*	1.7*
			(1.3–32.1)	(8.3–20.5)	(0.8–2.6)	(307.5–422.5)

The values represent the mean and range. RDS: rosettophyllous desert scrub; MDS: microphyllous desert scrub. The range is not shown for the *D. acerosa*, because there was only one analyzed compound sample. *P < 0.05 with regard to the reference site for the same species and vegetation type. Kamo: *K*. *mollis,* Agle:* A. lechuguilla*, Jadi: *J. dioica*, Dyac:* D. acerosa*, Latr: *L. tridentata*, Pain: *P. incanum*, and Ziac*: Z. acerosa.*

**Table 3:** Richness, total diversity, and by stratus for the RDS and the MDS.

Vegetation types		Contaminated		Reference	
		Richness	Diversity	Richness	Diversity
RDS	Herbaceous	10	0.75	10	0.61
	Bushy	10	0.71*	9	0.51
	Total	**20**	**1.03**	**19**	**0.82**

MDS	Herbaceous	8	0.6*	17	1.04
	Bushy	5	0.55*	15	0.98
	Subarboreal	2	0.05*	1	0
	Total	**13∗**	**0.88∗**	**32**	**1.28**

*P < 0.05.

**Table 4:** Composition of species for the rosettophyllous desert scrub (RDS) and the microphyllous desert scrub (MDS).

Site	Species	Acronym	RDS		MDS	
			Contaminated	Reference	Contaminated	Reference
Both sites	*Dyssodia acerosa *	Dyac	X	X	X	X
	*Prosopis laevigata *	Prla	—	—	X	X
	*Parthenium incanum *	Pain	—	—	X	X
	*Larrea tridentata *	Latr	—	—	X	X
	*Jatropha dioica *	Jadi	X	X	X	X
	*Zaluzania triloba *	Zatr	—	—	X	X
	*Senna wislizeni *	Sewi	—	—	X	X
	*Bahia absinthifolia *	Baab	—	—	X	X
	*Zinnia acerosa *	Ziac	—	—	X	X
	*Euphorbia prostrata *	Eupo	—	—	X	X
	*Tridens* sp.	Trsp	—	—	X	X
	*Buchloe dactyloides *	Buda	X	X	—	—
	*Agave lechuguilla *	Agle	X	X	—	—
	*Karwinskia mollis *	Kamo	X	X	—	—
	*Opuntia stenopetala *	Opst	X	X	—	—
	*Acalypha* sp.	Acsp	X	X	—	—
	*Mimosa biuncifera *	Mibi	X	X	—	—
	*Linum rupestre *	Liru	X	X	—	—

Only impacted	*Loeselia coerulea *	Loco	X	—	—	—
	*Menodora helianthemoides *	Mehe	X	—	—	—
	*Condalia fasciculata *	Cofa	X	—	—	—
	*Erigeron karvinskianus *	Erka	X	—	—	—
	*Euphorbia prostrata *	Eupo	X	—	—	—
	*Haplopappus spinulosus *	Hasp	X	—	—	—
	*Condalia* sp.	Cosp	X	—	X	—
	*Brickellia veronicaefolia *	Brbe	X	—	—	—
	*Lycium berlandieri *	Lybe	X	—	—	—
	*Scleropogon* sp.	Scsp	X	—	—	—
	*Tiquilia canescens *	Tica	X	—	X	—
	*Trixis angustifolia *	Tran	—	—	X	—

Only reference	*Bouteloua curtipendula *	Bocu	—	X	—	—
	*Cenchrus ciliaris *	Ceci	—	—	—	X
	*Kalanchoe blossfeldiana *	Kabl	—	—	—	X
	*Conyza schiedeana *	Cosh	—	—	—	X
	*Lycium berlandieri *	Lybe	—	—	—	X
	*Opuntia lindheimeri *	Opli	—	—	—	X
	*Physalis* sp.	Phsp	—	—	—	X
	*Menodora helianthemoides *	Mehe	—	—	—	X
	*Celtis pallida *	Cepa	—	—	—	X
	*Dalea bicolor *	Dabi	—	—	—	X
	*Erigeron karvisnkianus *	Erka	—	—	—	X
	*Opuntia leptocaulis *	Ople	—	—	—	X
	*Agave salmiana *	Agsa	—	—	—	X
	*Mimosa biuncifera *	Mibi	—	—	—	X
	*Buddleja scordioides *	Busc	—	—	—	X
	*Lippia* sp.	Lisp	—	—	—	X
	*Acalypha* sp.	Acsp	—	—	—	X
	*Cenchrus altianus *	Ceal	—	—	—	X
	*Lantana* sp.	Lasp	—	—	—	X
	*Salvia ballotaeflora *	Saba	—	X	—	X
	*Strenandum * sp.	Stsp	—	—	—	X
	*Viola* sp.	Visp	—	—	—	X
	*Bacopa procumbens *	Bapr	—	X	—	—
	*Evolvulus alsinoides *	Eval	—	X	—	—
	*Sida absintipholia *	Siab	—	X	—	—
	*Amelanchier denticulata *	Amde	—	X	—	—
	*Condalia mexicana *	Come	—	X	—	—
	*Dichondra sericea *	Dise	—	X	—	—
	*Ferocactus pilosus *	Fepi	—	X	—	—
	*Scutellaria potosina *	Scpo	—	X	—	—

**Table 5:** Captured species from the mining site in Villa de la Paz, San Luis Potosí.

Site	Family	Species	Acronym	Relative Abundance
Reference	Muridae	*Sigmodon hispidus *	Sihi	2.33
		*Neotoma mexicana *	Neme	2.33
		*Peromyscus maniculatus *	Pema	9.30
		*P. eremicus *	Peer	11.63
	Heteromyidae	*Chaetodipus nelsoni *	Chne	18.60
		*Dipodomys merriami *	Dime	55.81

Contaminated	Heteromyidae	*C. nelsoni *	Chne	47.06
		*D. merriami *	Dime	52.94

## References

[1] PNUMA/IPCS (1999). Evaluación de los riesgos químicos: humanos, ambientales y ecológicos. *Programa de las Naciones Unidad Para el Medio Ambiente (PNUMA)*.

[2] Nriagu JO, Pacyna JM (1988). Quantitative assessment of worldwide contamination of air, water and soils by trace metals. *Nature*.

[3] Madhavan N, Subramanian V (2000). Sulphide mining as a source of arsenic in the environment. *Current Science*.

[4] United Nations Environment Programme (UNEP) (2000). Mining and sustainable development II: challenges and perspectives. *Industry and Environment*.

[5] Fernández-Turiel JL, Aceñolaza P, Medina ME, Llorens JF, Sardi F (2001). Assessment of a smelter impact area using surface soils and plants. *Environmental Geochemistry and Health*.

[6] Jung MC (2001). Heavy metal contamination of soils and waters in and around the Imcheon Au-Ag mine, Korea. *Applied Geochemistry*.

[7] Morrey DR, Baker AJM, Cooke JA (1988). Floristic variation in plant communities on metalliferous mining residues in the northern and southern Pennines, England. *Environmental Geochemistry and Health*.

[8] Salemaa M, Vanha-Majamaa I, Derome J (2001). Understorey vegetation along a heavy-metal pollution gradient in SW Finland. *Environmental Pollution*.

[9] Koptsik S, Koptsik G, Livantsova S, Eruslankina L, Zhmelkova T, Vologdina Z (2003). Heavy metals in soils near the nickel smelter: chemistry, spatial variation, and impacts on plant diversity. *Journal of Environmental Monitoring*.

[10] Taucer E, Bernales M, Valdivia J (2006). Mitigación de las amenazas a la biodiversidad por las actividades mineras en el corredor de conservación Vilcabamba-Amboró (Bolivia-Perú). *Artesanos del socavón: Pequeña minería y minería artesanal en América Latina*.

[11] SGM (2012). *Anuario Estadístico de la Minería Mexicana Ampliada 2011*.

[12] Castro-Larragoitia J, Kramar U, Puchelt H (1997). 200 years of mining activities at La Paz/San Luis Potosí/Mexico—consequences for environment and geochemical exploration. *Journal of Geochemical Exploration*.

[13] Monroy M, Díaz-Barriga MF, Razo I, Carrizales L (2002). *Evaluación de la contaminación por arsénico y metales pesados (Pb, Cu, Zn) y análisis de riesgo en salud en Villa de la Paz-Matehuala, S.L.P. [M.S. thesis]*.

[14] Monroy M, Díaz-Barriga MF, Razo I, Carrizales L (2002). *Evidencias de contaminación de agua y sedimento por arsénico en el área de Cerrito Blanco, municipio de Matehuala, SLP [M.S. thesis]*.

[15] Razo I, Carrizales L, Castro J, Díaz-Barriga F, Monroy M (2004). Arsenic and heavy metal pollution of soil, water and sediments in a semi-arid climate mining area in Mexico. *Water, Air, and Soil Pollution*.

[16] Chiprés JA, Salinas JC, Castro-Larragoitia J, Monroy MG (2008). Geochemical mapping of major and trace elements in soils from the Altiplano Potosino, Mexico: a multi-scale comparison. *Geochemistry: Exploration, Environment, Analysis*.

[17] Chiprés JA, Castro-Larragoitia J, Monroy MG (2009). Exploratory and spatial data analysis (EDA-SDA) for determining regional background levels and anomalies of potentially toxic elements in soils from Catorce-Matehuala, Mexico. *Applied Geochemistry*.

[18] Yáñez L, Calderón J, Carrizales L, Díaz-Barriga MF, Díaz-Barriga F (1997). Evaluación del riesgo en sitios contaminados por plomo aplicando un modelo de exposición integral (IEUBK). *Evaluación de riesgos para la salud en la población expuesta a metales en Bolivia*.

[19] Calderón J, Navarro ME, Jiménez-Capdeville ME, Díaz-Barriga MF Neurobehavioral effects among children exposed chronically to arsenic, cadmium and lead.

[20] Carrizales L, Batres L, Ortiz M (1999). Efectos en salud asociados con la exposición a residuos peligrosos. *Scientiae Naturae*.

[21] Díaz-Barriga MF Metodología identificación y evaluación de riesgos para la salud en sitios contaminados.

[22] Mejía J, Yáñez L, Carrizales L, Díaz-Barriga MF (2001). Evaluación integral del riesgo en sitios contaminados: una propuesta metodológica. *Scientiae Naturae*.

[23] Yáñez L, García-Nieto E, Rojas E (2003). DNA damage in blood cells from children exposed to arsenic and lead in a mining area. *Environmental Research*.

[24] Gamiño-Gutiérrez SP, González-Pérez CI, Gonsebatt ME, Monroy-Fernández MG (2013). Arsenic and lead contamination in urban soils of Villa de la Paz (Mexico) affected by historical mine wastes and its effect on children’s health studied by micronucleated exfoliated cells assay. *Environmental Geochemistry and Health*.

[25] Jasso-Pineda Y, Espinosa-Reyes G, González-Mille D (2007). An integrated health risk assessment approach to the study of mining sites contaminated with arsenic and lead. *Integrated Environmental Assessment and Management*.

[26] Chapa-Vargas L, Mejía-Saavedra JJ, Monzalvo-Santos K, Puebla-Olivares F (2010). Blood lead concentrations in wild birds from a polluted mining region at Villa de la Paz, San Luis Potosí, Mexico. *Journal of Environmental Science and Health A*.

[27] Machado-Estrada B, Calderón J, Moreno-Sánchez R, Rodríguez-Zavala JS (2013). Accumulation of arsenic, lead, copper, and zinc, and synthesis of phytochelatins by indigenous plants of a mining impacted area. *Environmental Science and Pollution Research*.

[28] Hasan SA, Fariduddin Q, Ali B, Hayat S, Ahmad A (2009). Cadmium: toxicity and tolerance in plants. *Journal of Environmental Biology*.

[29] Eisler R (1998). *Copper Hazards to Fish, Wildlife, and Invertebrates: A Synoptic Review*.

[30] Efroymson RA, Will ME, Suter GW, Wooten AC (1997). *Toxicological Benchmarks for Screening Contaminants of Potential Concern for Effects on Terrestrial Plants: 1997 Revision*.

[31] Cheng S (2003). Heavy metals in plants and phytoremediation. *Environmental Science and Pollution Research*.

[32] Kashem A, Singh BR (1999). Heavy metal contamination of soil and vegetation in the vicinity of industries in Bangladesh. *Water, Air, and Soil Pollution*.

[33] Peplow D Environmental impacts of mining in eastern Washington.

[34] Medellín RA, Equihua M, Amin MA (2000). Bat diversity and abundance as indicators of disturbance in neotropical rainforest. *Conservation Biology*.

[35] Mishra BP, Tripathi OP, Tripathi RS, Pandey HN (2004). Effects of anthropogenic disturbance on plant diversity and community structure of a sacred grove in Meghalaya, northeast India. *Biodiversity and Conservation*.

[36] Vásquez JA, Vega JMA, Matsuhiro B, Urzúa C (1999). The ecological effects of mining discharges on subtidal habitats dominated by macroalgae in northern Chile: population and community level studies. *Hydrobiologia*.

[37] Niyogi DK, Lewis WM, McKnight DM (2002). Effects of stress from mine drainage on diversity, biomass, and function of primary producers in mountain streams. *Ecosystems*.

[38] Ramadan AA (2003). Heavy metal pollution and biomonitoring plants in Lake Manzala, Egypt. *Pakistan Journal of Biological Sciences*.

[39] Sullivan TP, Sullivan DS (2003). Vegetation management and ecosystem disturbance: impact of glyphosate herbicide on plant and animal diversity in terrestrial systems. *Environmental Reviews*.

[40] Fountain MT, Hopkin SP (2004). Biodiversity of collembola in urban soils and the use of *Folsomia candida* to assess soil ‘quality’. *Ecotoxicology*.

[41] Freitas H, Prasad MNV, Pratas J (2004). Plant community tolerant to trace elements growing on the degraded soils of São Domingos mine in the south east of Portugal: environmental implications. *Environment International*.

[42] Talmage SS, Walton BT (1991). Small mammals as monitors of environmental contaminants. *Reviews of Environmental Contamination and Toxicology*.

[43] Erry BV, MacNair MR, Meharg AA, Shore RF (2000). Arsenic contamination in wood mice (*Apodemus sylvaticus*) and bank voles (*Clethrionomys glareolus*) on abandoned mine sites in southwest Britain. *Environmental Pollution*.

[44] Torres KC, Johnson ML (2001). Bioaccumulation of metals in plants, arthropods, and mice at a seasonal wetland. *Environmental Toxicology and Chemistry*.

[45] Milton A, Cooke JA, Johnson MS (2003). Accumulation of lead, zinc, and cadmium in a wild population of *Clethrionomys glareolus* from an abandoned lead mine. *Archives of Environmental Contamination and Toxicology*.

[46] Ieradi LA, Zima J, Allegra F (2003). Evaluation of genotoxic damage in wild rodents from a polluted area in the Czech Republic. *Folia Zoologica*.

[47] Šumbera R, Baruš V, Tenora F (2003). Heavy metals in the silvery mole-rat, *Heliophobius argenteocinereus* (Bathyergidae, Rodentia) from Malawi. *Folia Zoologica*.

[48] Reynolds H (1958). The ecology of the Merriam kangaroo rat (*Dipodomys merriami* Mearns) on the grazing lands of southern Arizona. *Ecological Monographs*.

[49] da Silva J, de Freitas TRO, Marinho JR, Speit G, Erdtmann B (2000). An alkaline single-cell gel electrophoresis (comet) assay for environmental biomonitoring with native rodents. *Genetics and Molecular Biology*.

[50] Festa F, Cristaldi M, Ieradi LA, Moreno S, Cozzi R (2003). The comet assay for the detection of DNA damage in *Mus spretus* from Doñana National Park. *Environmental Research*.

[51] Kataev GD, Suomela J, Palokangas P (1994). Densities of microtine rodents along a pollution gradient from a copper-nickel smelter. *Oecologia*.

[52] García E (2004). *Modificaciones al Sistema de Clasificación Climática de Köppen*.

[53] Instituto Nacional de Estadística, Geografía e Informática (INEGI) (2002). *Síntesis de información geográfica del estado de San Luis Potosí*.

[54] Rzedowski J (1978). *Vegetación de México*.

[55] Instituto Nacional de Estadística, Geografía e Informática (INEGI) (2012). *Anuario Estadístico: San Luis Potosí*.

[56] Molina MC, García ME, Aguirre RR, González CE (1991). La reserva de semillas de un pastizal de *Bouteloua gracilis*. *Agrociencia*.

[57] Mueller-Dombois D, Ellengerg H (1974). *Aims and Methods of Vegetation Ecology*.

[58] Franco LJ, de la Cruz GA, Cruz AG (1989). *Manual de Ecología*.

[59] Brower JE, Zar JH, von Ende CN (1998). *Field and Laboratory Methods for General Ecology*.

[60] Seber GAF (1973). *The Estimation of Animal Abundance and Related Parameters*.

[61] Boitani L, Fuller TK (2000). *Research Techniques in Animal Ecology: Controversies and Consequences*.

[62] Romero-Almaráz M, Sánchez-Hernández C, García-Estrada C, Owen R (2007). *Mamíferos pequeños: Manual de técnicas de captura, preparación, preservación y estudio*.

[63] Magurran AE (1989). *Diversidad ecológica y su medición*.

[64] Margalef R (1981). *Ecología*.

[65] Hutcheson K (1970). A test for comparing diversities based on the Shannon formula. *Journal of Theoretical Biology*.

[66] Clements WH, Newman MC (2002). *Community Ecotoxicology*.

[68] Cervantes C, Moreno SR (1999). *Contaminación ambiental por metales pesados: impacto en los seres vivo*.

[69] Kabata-Pendias A, Pendias H (2001). *Trace Elements in Soils and Plants*.

[70] Lorenz SE, Hamon RE, Holm PE (1997). Cadmium and zinc in plants and soil solutions from contaminated soils. *Plant and Soil*.

[71] Memon AR, Aktoprakligil D, Özdemir A, Vertii A (2001). Heavy metal accumulation and detoxification mechanisms in plants. *Turkish Journal of Botany*.

[72] Ghosh M, Singh SP (2005). A review on phytoremediation of heavy metals and utilization of its byproducts. *Applied Ecology and Environmental Research*.

[73] Youngman AL, Williams TL, Tien LS Patterns of accumulation of heavy metals in non-woody vegetation established on zinc-lead smelter contaminated soils.

[74] Lumsdon DG, Meeussen JCL, Paterson E, Garden LM, Anderson P (2001). Use of solid phase characterisation and chemical modelling for assessing the behaviour of arsenic in contaminated soils. *Applied Geochemistry*.

[75] Wang Q-R, Cui Y-S, Liu X-M, Dong Y-T, Christie P (2003). Soil contamination and plant uptake of heavy metals at polluted sites in China. *Journal of Environmental Science and Health A*.

[76] Malaisse F, Baker A, Ruell S (1999). Diversity of plant communities and leaf heavy metal content at Luiswishi copper/cobalt mineralization, Upper Katanga, Dem. Rep. Congo. *Biotechnology Agronomy Society Environment*.

[77] Luoma SN (1977). The dynamics of biologically available mercury in a small estuary. *Estuarine and Coastal Marine Science*.

[78] Martin HW, Young TR, Kaplan DI, Simon L, Adriano DC (1996). Evaluation of three herbaceous index plant species for bioavailability of soil cadmium, chromium, nickel and vanadium. *Plant and Soil*.

[79] Gardea-Torresdey JL, Polette L, Arteaga S, Tiemann KJ, Bibb J, Gonzalez JH Determination of the content of hazardous heavy metals on Larrea tridentata grown around a contaminated area.

[80] Gardea-Torresdey JL, Bibb J, Tiemann KJ, González JH, Arenas JL Adsorption of copper ions from solution by heavy metal stressed Larrea tridentate (creosote bush) biomass.

[81] Bu-Olayan AH, Thomas BV (2002). Biomonitoring studies on the lead levels in mesquite (*Prosopis juliflora*) in the arid ecosystem of Kuwait. *Kuwait Journal of Science and Engineering*.

[82] Sienkiewicz J (1986). Effect of heavy-metals industry on plant communities. *The Science of the Total Environment*.

